# Crosstalk Between Abnormal TSHR Signaling Activation and PTEN/PI3K in the Dedifferentiation of Thyroid Cancer Cells

**DOI:** 10.3389/fonc.2021.718578

**Published:** 2021-09-28

**Authors:** Fang Feng, Huiqin Han, Shuqi Wu, Hui Wang

**Affiliations:** ^1^ Department of Nuclear Medicine, Xinhua Hospital, Shanghai Jiao Tong University, School of Medicine, Shanghai, China; ^2^ Shanghai Mental Health Center, Shanghai Jiao Tong University, School of Medicine, Shanghai, China

**Keywords:** differentiated thyroid carcinoma, thyrotropin receptor, radioiodine, dedifferentiation, migration

## Abstract

Iodide uptake and the metabolism of thyroid cells are regulated by thyrotropin (TSH)-TSH receptor (TSHR) signaling. Thus, it is necessary to elevate serum TSH levels by T4 withdraw or rTSH administration to facilitate radioiodide (^131^I) therapy for differentiated thyroid cancer (DTC). However, non-iodide-avid metastases of DTC which is dedifferentiated do not respond to stimulation by high levels of TSH, suggesting abnormal TSH-TSHR signal transduction in cancer cells. In addition, PI3K/AKT/mTOR signaling activation has been shown to be associated with the dedifferentiated phenotype of thyroid cancer, but the mechanism remains elusive. Therefore, in this study, we aimed to explore the role of abnormal TSH-TSHR signaling activation in regulating iodide uptake and cell mobility in thyroid cancer and its relationship with PI3K/AKT/mTOR signaling. We found that in thyroid cancer cells, TSH binds TSHR coupled to the Gα_12/13_ protein and then activates RhoA through interacting with leukemia associated RhoA guanine exchange factor (LARG). This results in a promigration tumorigenic phenotype independent of canonical TSHR-Gα_S_ signaling that regulates the expression of molecules involved in iodine uptake and metabolism. We observed that signaling pathways downstream of Gα_12/13_ signaling were increased, while that of Gα_s_ signaling was decreased in thyroid cancer cells undergoing dedifferentiation compared to control cells following stimulation with different levels of TSH. PI3K/AKT/mTOR signaling activation enhanced Gα_12/13_ signaling through increasing LARG levels but also inhibited the expression of molecules downstream of Gα_s_ signaling, including thyroid-specific molecules, and iodide uptake. In summary, our results demonstrate the noncanonical activation of TSH-TSHR signaling and its role in increasing the cell mobility and dedifferentiation of thyroid cancer through crosstalk with PI3K/AKT/mTOR signaling.

## Introduction

Thyroid cancer, over 90% of which is differentiated thyroid cancer (DTC), is one of the most rapidly increasing cancers worldwide. Although most patients with DTC show good prognosis and are cured by thyroidectomy followed by radioiodine (^131^I) ablation, 1-4% of patients at initial diagnosis and 7-23% of patients during follow-up develop distant metastasis (1, 2). To date, ^131^I therapy remains the first-line treatment for metastatic DTC, and a high level of ^131^I uptake is a marker of good prognosis (3, 4). However, in one-third of metastatic DTC patients, the cancer cells undergo dedifferentiation and lose their ability to accumulate ^131^I, with increased cell proliferation and invasion; therefore, these patients exhibit a poorer prognosis (3–6). In addition, ^131^I treatment is limited in thyroid cancer that is poorly differentiated or anaplastic thyroid cancer (ATC), which do not take up or take up very low levels of ^131^I (7), but the molecular mechanism remains elusive.

Iodide uptake and thyroid hormone (TH) biosynthesis, vital functions of thyroid cells, are mainly controlled by thyrotropin (TSH) and its receptor (TSHR). As a G protein coupled receptor (GPCR), TSHR can couple with all four G protein families to activate downstream signaling (8), though the Gα_s_-adenylyl cyclase-cyclic AMP (cAMP) and Gα_q_-calcium pathways have been reported to be the most physiologically relevant (9, 10). Classically, through coupling to Gα_s_ and activating the cAMP-PKA pathway, TSH-TSHR stimulates the expression of various thyroid-specific genes involved in iodide metabolism, such as thyroglobulin (Tg), thyroperoxidase (TPO) and sodium-iodide symporter (NIS), in turn increasing the iodide uptake of normal or tumorous thyroid cells. Therefore, T_4_ withdrawal or rTSH administration is routinely utilized to increase serum TSH levels to facilitate ^131^I therapy for DTC patients. However, in patients with non-iodide-avid metastases, high levels of TSH cannot stimulate iodide uptake by the lesions. Our previous work has shown that TSHR expression levels play important roles in maintaining the differentiation of thyroid cancer cells (11–13). Nevertheless, in some cases, TSHR expression has been reported to remain normal (14). Moreover, few loss-of-function TSHR mutations in DTC tissue have been reported. Therefore, we hypothesized that the abnormal transduction of downstream TSHR signaling contributes to decreased iodide uptake and is related to the invasion phenotype.

Among G proteins, Gα_12/13_ is closely associated with increased cell mobility in some cancer tissues (15), but its role in TSHR signaling in thyroid cancer remains elusive. In addition, crosstalk between PI3K/AKT/mTOR signaling activation and multiple protumorigenic pathways has been well established in multiple cancer models, including thyroid cancers, and shown to play important roles in cancer initiation and progression (16). Importantly, PI3K/AKT/mTOR signaling activation is associated with reduced NIS expression and iodide uptake in thyroid cancer cells (17). These findings suggest that PI3K signaling is involved in crosstalk with TSHR signaling, which affects the differentiation of thyroid cancer cells. We therefore hypothesized that in addition to its canonical function in regulating iodide uptake and TH synthesis, the abnormal activation of TSHR signaling, such as that through Gα_12/13_ downstream signaling, promotes tumorigenesis and is associated with the dedifferentiation of DTC cells.

## Materials and Methods

### Cell Lines and Culture Conditions

We utilized human thyroid cancer cell lines TPC1, BCPAP and FTC-133, grown in DMEM, RPMI-1640 and DMEM/F12 medium respectively, supplemented with 10% fetal bovine serum (FBS) and 1% penicillin/streptomycin (P/S). We also used a rat thyroid cell line PCCL3, cultured in Coon’s Modified F-12 medium (Sigma T8931), supplemented with 1% P/S, 5% FBS, 10mM NaHCO_3_, 2mM L-Glutamine, 1mU/ml TSH (Sigma I1882), 10μg/ml bovine insulin (Sigma), 10nM Hydrocortisone (Sigma H4001), 10ng/ml Somatostatin (Sigma S1763), 5μg/ml Transferrin (Sigma T8158) and 2 ng/ml L-Glycyl-Histidyl-Lysine (Sigma G1887). For wound healing assay, HBSS was used instead of FBS in the medium. All cell lines were maintained at 37°C and 5% CO_2_ culture conditions. All experiments were conducted with cells at passage numbers between 8 and 15. All cell lines were authenticated through the American Type Culture Collection (ATCC) human cell authentication service (ATCC^®^ 135-XV™) and were 100% matched to the reported STR profiles in the DSMZ database (test date 19/08/2018).

### Reagents

Rapamycin, Y27632 were purchased from Sigma. LY294002 and MK-2206 were obtained from Selleckchem (Houston, TX). Dimethyl sulfoxide (DMSO, Sigma) served as the vehicle control for the experiments involving PI3K/AKT/mTOR inhibitor treatment.

### RNA Extraction and qRT-PCR

RNA was extracted from the cell lines using the NucleoSpin^®^ RNA Plus kit (MACHEREY-NAGEL GmbH & Co. KG, D̈ren, Germany) following the user manual, and reverse transcribed using Superscript III reverse transcriptase (Life Technologies). Primers were designed for gene transcripts of interest and cDNA quantified using SYBR Green (Life Technologies). We utilized the Applied Biosystems 7500 Real-Time PCR System. Results were analyzed using the standard ΔΔCT method.

### Immunoblotting

Protein was extracted from whole cell lysates using the Mammalian Protein Extraction Reagent M-PER (Thermo Scientific Pierce, Rockford, IL) supplemented with a cocktail of protease and phosphatase inhibitors (Sigma) and quantified through the BCA protein assay (Thermo Scientific Pierce). Lysates were separated by SDS-PAGE and transferred onto nitrocellulose membranes. We probed for anti-TSHR (4C1) mouse monoclonal (Thermo Fisher Scientific) at 1:1000, anti-Gα_12_ (N3C3) rabbit polyclonal (GeneTex, Irvine, CA) at 1:1000, anti-Gα_13_ rabbit polyclonal (NewEast Bioscience, Malvern, PA) at 1:1000, anti-PTEN (6H2.1) mouse monoclonal (Cascade Bioscience, Winchester, MA) at 1:1000, anti-LARG mouse monoclonal (EMD Millipore, Temecula, CA) at 1:10000, anti Phospho-AKT (Ser473) rabbit polyclonal (Cell Signaling #9271L) at 1:1000, anti-Phospho-p70 S6 Kinase (Thr389) (1A5) mouse monoclonal (Cell Signaling #9206S) at 1:1000, anti Phospho-S6 ribosomal protein (S235/236) rabbit monoclonal (Cell Signaling #4858S) at 1:1000, anti-Phospho-cofilin (Abcam #ab12866) at 1:1000 and anti-GAPDH rabbit monoclonal (Cell Signaling #2118) at 1:20000 dilution. Blots were scanned digitally using the ChemiDoc™ touch imaging system (Bio-Rad, Hercules, CA). Densitometry was performed using ImageJ software.

### Tg Radioimmunoassay

Thyroglobulin levels in thyroid cancer cells were measured using radioimmunoassay according to the manufacturer’s instructions as described previously (11). Total proteins were extracted from thyroid cancer cells as described above and quantified by using the BCA method. Relative Tg content was calculated as the ratio of Tg concentration to total protein concentration.

### Plasmid Construction and Transfection

cDNAs encoding the constitutively active Gα_12_ (Gα_12_Q229L) and Gα_13_ (Gα_13_Q226L) were subcloned into pCMV5 vector. Cells at 70% confluence were transiently transfected with plasmid using Lipofectamine 3000 reagent (Invitrogen, Carlsbad, CA) according to the manufacturer’s instructions. The efficiency of transfection was determined using Western blotting and RT-PCR to evaluate protein and mRNA expression following cell collection. For PTEN transfection, we used GFP-tagged wild type *PTEN* plasmid (pcDNA3.1 vector). The empty vector pcDNA3.1 was set as the control. For transient overexpression, cell lines were transfected with Lipofectamine 3000 reagent (Invitrogen, Carlsbad, CA) according to the manufacturer’s instructions. Cells were grown for at least 48 hours before harvesting. To generate stable cell lines, retrovirally-transduced cells were kept under 1 µg/ml Puromycin selection, and the different Puromycin-resistant cell colonies were pooled together for downstream interrogation.

### RhoA Pull-Down Activation Assay

Rho activity was measured using the RhoA pull-down activation assay kit (Cytoskeleton, Denver, CO). Cells were homogenized in the cell lysis buffer containing protease inhibitor cocktail. Lysates were cleared by centrifugation and the soluble fraction was incubated with 50μg of GST-Rhotekin beads, which can specifically pull down activated RhoA, for 1 hour at 4°C. Beads were collected by centrifugation for 1minute at 3,000×g and 4°C, washed, resuspended in Laemmli buffer, separated by SDS-PAGE, and then analyzed by western blotting using a mouse monoclonal anti-RhoA antibody (1:500 dilution). Relative variations in normalized RhoA-GTP level is defined as the ratio of the level of activated GTP-bound forms of RhoA and the level of total RhoA in NIS stable cells, divided by the same ratio in control cells.

### Immunoprecipitation

Cells were pelleted and lysed with M-PER (Thermo Scientific Pierce) supplemented with a cocktail of protease and phosphatase inhibitors (Sigma-Aldrich). Protein lysates were collected by centrifugation at 13,000 RPM for 10 min at 4°C and pre-cleared by incubation with Thermo Protein A/G Dynabeads (Thermo Scientific Pierce) for 3 hours at 4°C on a rotator. Pre-cleared protein lysates were quantified with the BCA Protein Assay Kit (Thermo Scientific Pierce), and 2 mg/ml lysates were prepared. We used anti-Gα_12/13_, anti-LARG and anti-RhoA antibodies for pull-down and immunoblotting at the recommended dilutions. Cell lysates were separated by SDS-PAGE and transferred onto nitrocellulose membranes. Blots were scanned digitally using the ChemiDoc™ touch imaging system (Bio-Rad).

### Knockdown of TSHR, Gα_12/13_, LARG, or PTEN Through siRNA

Thyroid cancer cells were seeded in 6 well plates and allowed to grow overnight. For TSHR, Gα_12/13_, LARG or PTEN knockdown, cells were transfected with TSHR, Gα_12/13_, LARG or PTEN siRNA smartpool (Dharmacon, Lafayette, CO) using Lipofectamine RNAiMAX (Thermo Fisher Scientific) according to the manufacturer’s instructions. We used ON-TARGET non-targeting siRNA pool (Dharmacon) as a control (siNT). Cells were collected for downstream analysis from 48 hours to 72 hours after knockdown transfection. We used Western blot and qRT-PCR analysis to confirm knockdown.

### Immunofluorescence

Cells were seeded on coverslips and were fixed with 4% paraformaldehyde for 2 minutes at room temperature, washed with PBS, and then incubated with 1% Triton X-100 for 2 minutes at room temperature. Cells were blocked with 10% goat serum (Vector Laboratories Inc, Burlingame, CA) for 1 hour at room temperature, and incubated with rabbit anti-LARG antibodies (at 1:500 dilution) overnight at 4°C. Secondary antibodies (Alexa 568) were incubated at 1:2000 dilution for 1 hour in the dark at room temperature. Coverslips were mounted using ProLong Gold Antifade mountant with DAPI (Invitrogen). Slides were visualized and images obtained using the Leica TCS SP5 II confocal microscope (Leica Microsystems, Heidelberg, Germany).

### Measurement of Intracellular cAMP Levels

Intracellular cAMP levels were measured follow the method reported previously (18). Briefly, culture medium was removed 48 h after transfection or treatment with drugs and replaced by HEPES buffer for 30 min. Then, the medium was discarded and replaced with 0.1 M HCl. The cell extracts were dried in a vacuum concentrator, resuspended in water, and diluted appropriately for cAMP measurements by RIA. Duplicate samples were assayed in all experiments. Results are expressed as picomoles cAMP per milliliter. Experiments were performed in triplicates.

### *In Vitro* Migration Assay

For wound-healing assays, confluent-transfected cells monolayers were scraped with a fine sterile pipette tip, placed in the cell incubator (5% CO_2_, 37°C). Image were acquired at 0 and 20 hours after scraping using a Leica DMI3000B microscope (Leica Microsystems). HBSS was used instead of FBS in the medium for suppressing cell proliferation. For migration inhibitor tests, Rho-associated coiled-coil kinase (ROCK) inhibitor Y-27632 at 30μM was added at 1 hour before scraping. The wound margin area was determined by image processing using ImageJ software. The average wound-healing speeds were calculated. Each condition was run in triplicate. Data represent means ± SEM from 3 independent experiments.

### Radioiodide Uptake Assay

RAIU assay was performed follow the methods reported previously (19). Briefly, cells were pretreated with siRNA for 24h before the assay. At 24 h, cells were incubated with 2 μCi Na^125^I in 5 μM nonradioactive NaI for 30 min at 37°C with 5% CO2. Cells were then washed with cold Hank’s Balanced Salt Solution two times and then lysed in cold 95% ethanol for 20 min at room temperature. Cell lysate was collected and counted for radioactivity by the gamma-counter. RAIU not mediated by NIS was excluded by performing the assay in the presence of 3 mM perchlorate $\left(ClO_{4}^{-}\right)$ , a competitive inhibitor of iodide uptake by NIS. Experiments were performed in triplicates.

### Statistical Analyses

Experimental data between control and experimental cells are given as means ± SEM with n corresponding to the number of experiments performed. The Student’s *t* test was used for significance testing as indicated in figure legends. All statistical tests were two-sided, and p-values <0.05 were deemed significant.

## Results

### Activation of TSH-TSHR Signaling Increases the Migration of Thyroid Cancer Cells *In Vitro*

**Figure 1:**
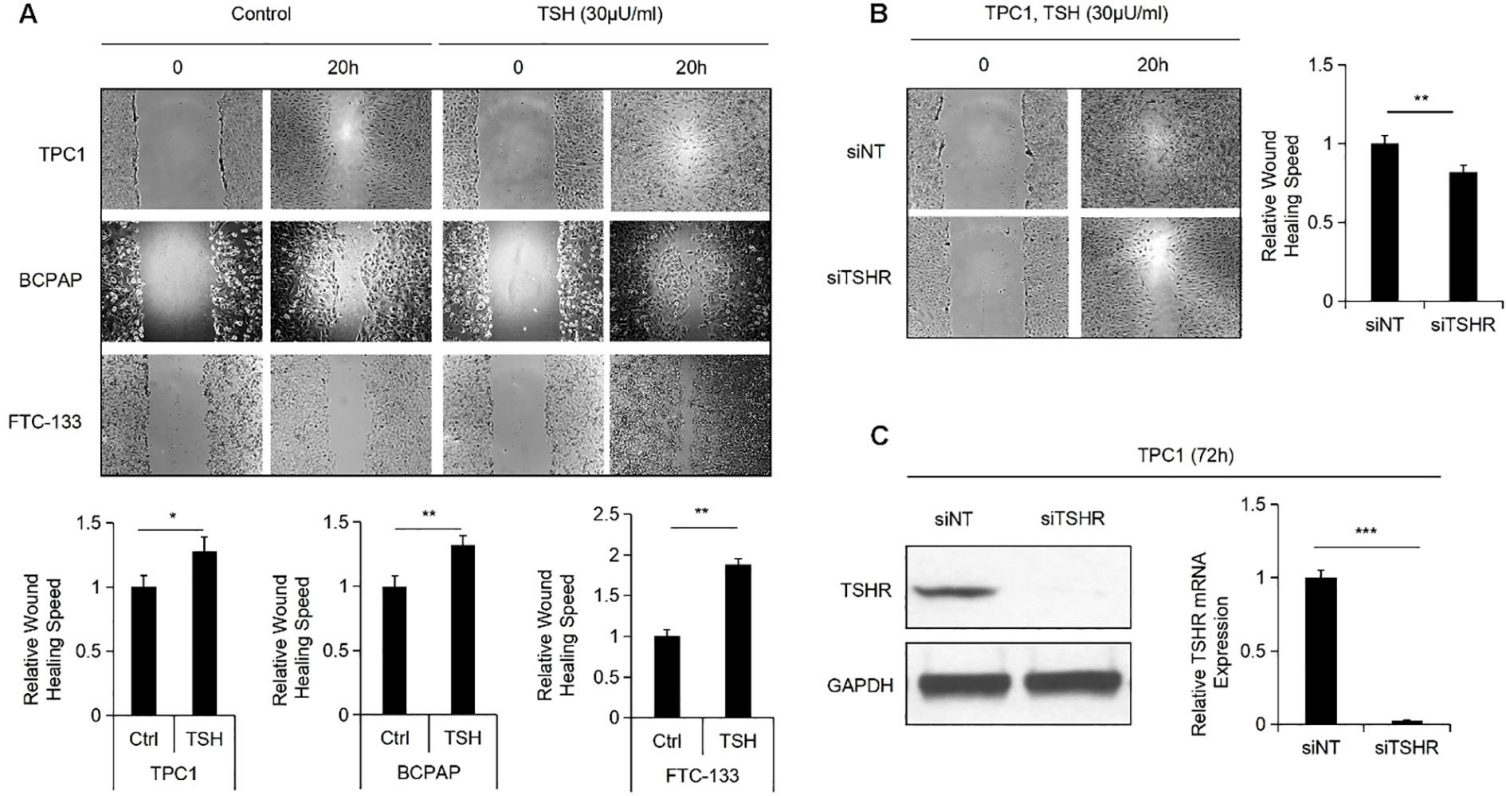
TSH increasing RhoA activation and cell migration is Gα12/13 dependent. **(A)** Immunoblots of RhoA and GAPDH from total cell lysates and RhoA after GST-Rhotekin pull-down (RhoA-GTP form) in TPC1, BCPAP and FTC-133 cells. Bar graphs, normalized ratios of active RhoA relative to total RhoA levels. Data represent means ± SEM of 3 independent experiments. **(B)** Wound-healing assay for TPC1 cells with treatment of 30 μU/mL TSH and 30μM Y27632 or control. Bar graph Data represents relative wound healing rate of TPC1 cells with treatment of 30 μU/mL TSH and 30μM Y27632 normalized with control. Immunoblots of phosphor-coffin and GAPDH in TPC1 cells incubated with 30 μU/mL TSH and 30μM Y27632 for 72 hours. and Data represent means ± SEM of 3 independent experiments. **(C)** Immunoblots of RhoA, Gα12 and GAPDH from total cell lysates and RhoA after GST-Rhotekin pull-down (RhoA-GTP form) in TPC1, BCPAP and FTC-133 transiently transfected with non-targeting siRNA (siNT) or Gα12 and Gα13 siRNA (si Gα12/13) with treatment of 30μU/mL TSH. GAPDH is used as a loading control for normalization. The bar graph represents normalized ratios of active RhoA relative to total RhoA levels. **(D)** Immunoblots of RhoA, Gα12 and GAPDH from total cell lysates and RhoA after GST-Rhotekin pull-down in TPC1 stably transfected with Gα12WT or Gα12Q229L. Bar graph, normalized ratios of active RhoA relative to total RhoA levels. *P 0.05, **P 0.01, ***P 0.001.

TSH canonically regulates the functions of thyroid cells through binding TSHR, which then activates downstream signaling. To test our hypothesis that TSH-TSHR signaling is also associated with the cell migration phenotype in thyroid cancer cells, we performed a wound healing assay in the TPC1, BCPAP and FTC-133 thyroid cancer cell lines grown in medium containing 30 μU/ml TSH, which was observed to induce sufficient TSHR expression (**Supplementary Figure S1**). HBSS was used instead of FBS in the medium for suppressing cell proliferation. The cell migration rate was increased in all of the three cell lines after their incubation with TSH. The migration rate was increased 1.3-, 1.4- and 1.8-fold in TPC1, BCPAP and FTC-133 cells, respectively, after treatment with TSH compared to the migration rate of corresponding control cells (*P*=0.016, 0.004 and 0.003, respectively; **Figure 1A**). To further validate that this promigration effect occurs through TSHR signaling, we transiently knocked down TSHR in thyroid cancer cells through siRNA. These cells were cultured with 30 μU/ml TSH, after which we compared their migration rates. The migration rates of TPC1, BCPAP and FTC-133 cells at 72 h following TSHR siRNA knockdown were reduced by approximately 20%, 23% and 25%, respectively, compared to those in control cells transfected with siNT (*P*=0.007, 0.005 and 0.004; **Figure 1B** and **Supplementary Figure S2**). siRNA-mediated TSHR knockdown was confirmed by Western blotting and qRT-PCR (**Figure 1C** and **Supplementary Figure S2**). Taken together, these data provide evidence that TSH-TSHR signaling activation increases thyroid cancer cell migration.

### TSH-TSHR Increases RhoA Activation in Thyroid Cancer Cells Through Gα_12/13_

**Figure 2:**
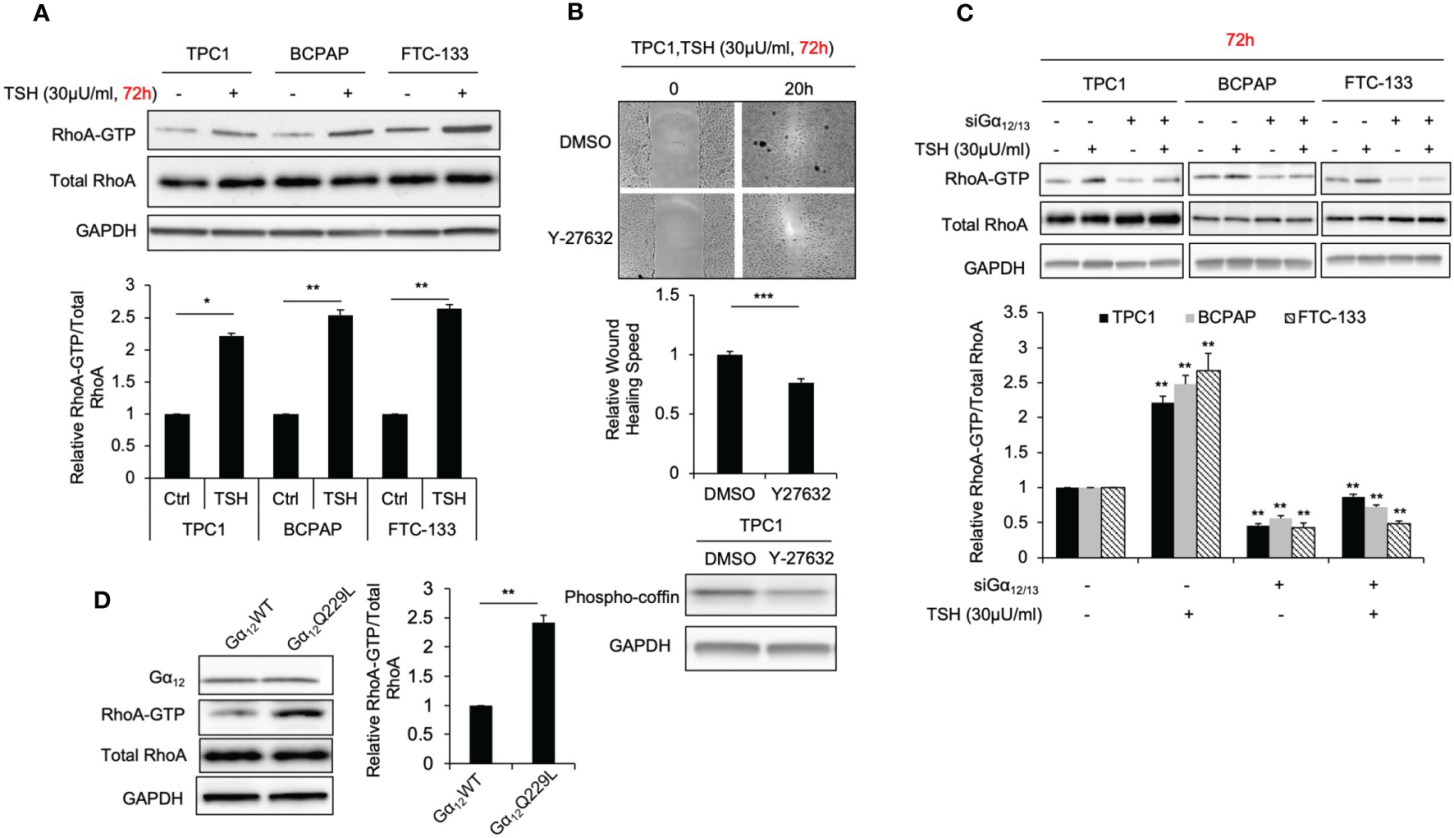
TSH increasing RhoA activation and cell migration is Gα_12/13_ dependent. **(A)** Immunoblots of RhoA and GAPDH from total cell lysates and RhoA after GST-Rhotekin pull-down (RhoA-GTP form) in TPC1, BCPAP and FTC-133 cells. Bar graphs, normalized ratios of active RhoA relative to total RhoA levels. Data represent means ± SEM of 3 independent experiments. **(B)** Wound-healing assay for TPC1 cells with treatment of 30 μU/mL TSH and 30μM Y27632 or control. Bar graph Data represents relative wound healing rate of TPC1 cells with treatment of 30 μU/mL TSH and 30μM Y27632 normalized with control. Immunoblots of phosphor-coffin and GAPDH in TPC1 cells incubated with 30 μU/mL TSH and 30μM Y27632 for 72 hours. and Data represent means ± SEM of 3 independent experiments. **(C)** Immunoblots of RhoA, Gα_12_ and GAPDH from total cell lysates and RhoA after GST-Rhotekin pull-down (RhoA-GTP form) in TPC1, BCPAP and FTC-133 transiently transfected with non-targeting siRNA (siNT) or Gα_12_ and Gα_13_ siRNA (si Gα_12/13_) with treatment of 30μU/mL TSH. GAPDH is used as a loading control for normalization. The bar graph represents normalized ratios of active RhoA relative to total RhoA levels. **(D)** Immunoblots of RhoA, Gα_12_ and GAPDH from total cell lysates and RhoA after GST-Rhotekin pull-down in TPC1 stably transfected with Gα_12_WT or Gα_12_Q229L. Bar graph, normalized ratios of active RhoA relative to total RhoA levels. ^*^ *P* < 0.05, ^**^ *P* < 0.01, ^***^ *P* < 0.001.

RhoA activation plays an important role in thyroid cancer cell motility, according to our most recent study (20). Hence, as the downstream activation of RhoA was observed to be relevant to the migration phenotype, we observed its effects in thyroid cancer cells treated with TSH with a GST-Rhotekin pulldown assay. The relative levels of the activated GTP-bound form of RhoA in TPC1, BCPAP and FTC-133 cells grown with 30 μU/ml TSH for 72 hours were significantly higher than those in control cells (2.2-, 2.5- and 2.6-fold; *P*=0.018, 0.009 and 0.001, respectively; **Figure 2A**). Furthermore, the Rho-associated kinase (ROCK) inhibitor Y-27632, which was conformed to decrease phospho-cofilin by immunoblot, suppressed the migration rate of thyroid cancer cells treated with TSH in the absent of FBS. In TPC1 cells, the migration rate was decreased 0.77-fold after treatment with Y-27632 compared to that in control cells (*P*<0.001, **Figure 2B**). These data further support the involvement of the RhoA-ROCK pathway in enhanced cell migration downstream of TSH-TSHR signaling.

As a GPCR, TSHR can couple the members of four G protein families to activate downstream signaling. Among these G protein family members, Gα_12/13_ has been reported to be associated with the migration phenotype (21). To investigate whether Gα_12/13_ is involved in the mechanism by which TSHR signaling regulates RhoA activation and enhances cell migration, we measured the relative RhoA-GTP levels in TPC1, BCPAP and FTC-133 cells transiently transfected with Gα_12_ and/or Gα_13_ siRNA or siNT treated with TSH or control. Gα_12_ and Gα_13_ knockdown was confirmed by Western blotting (**Supplementary Figure S3**). Again, we observed that cells treated with TSH showed high levels of RhoA-GTP. After Gα_12/13_ knockdown, this increase in RhoA-GTP levels was abrogated (**Figure 2C**). To further confirm that Gα_12/13_ is involved in RhoA activation, we transiently and stably transfected TPC1 cells with plasmid encoding active Gα_12_Q229L or Gα_13_Q226L mutant and then compared the RhoA-GTP levels (22). The RhoA-GTP level was increased 2.3-fold (*P*=0.005) in TPC1 cells stably transfected with Gα_12_Q229L plasmid compared to that in the control cells transfected with Gα_12_WT plasmid (**Figure 2D**). Similar results were observed in cells transfected with Gα_13_Q226L plasmid. Taken together, these data show that TSH-TSHR enhances RhoA activation and downstream cellular migration through Gα_12/13_.

### Gα_12/13_ Activates RhoA Through Its Interaction With LARG in Thyroid Cancer Cells

**Figure 3:**
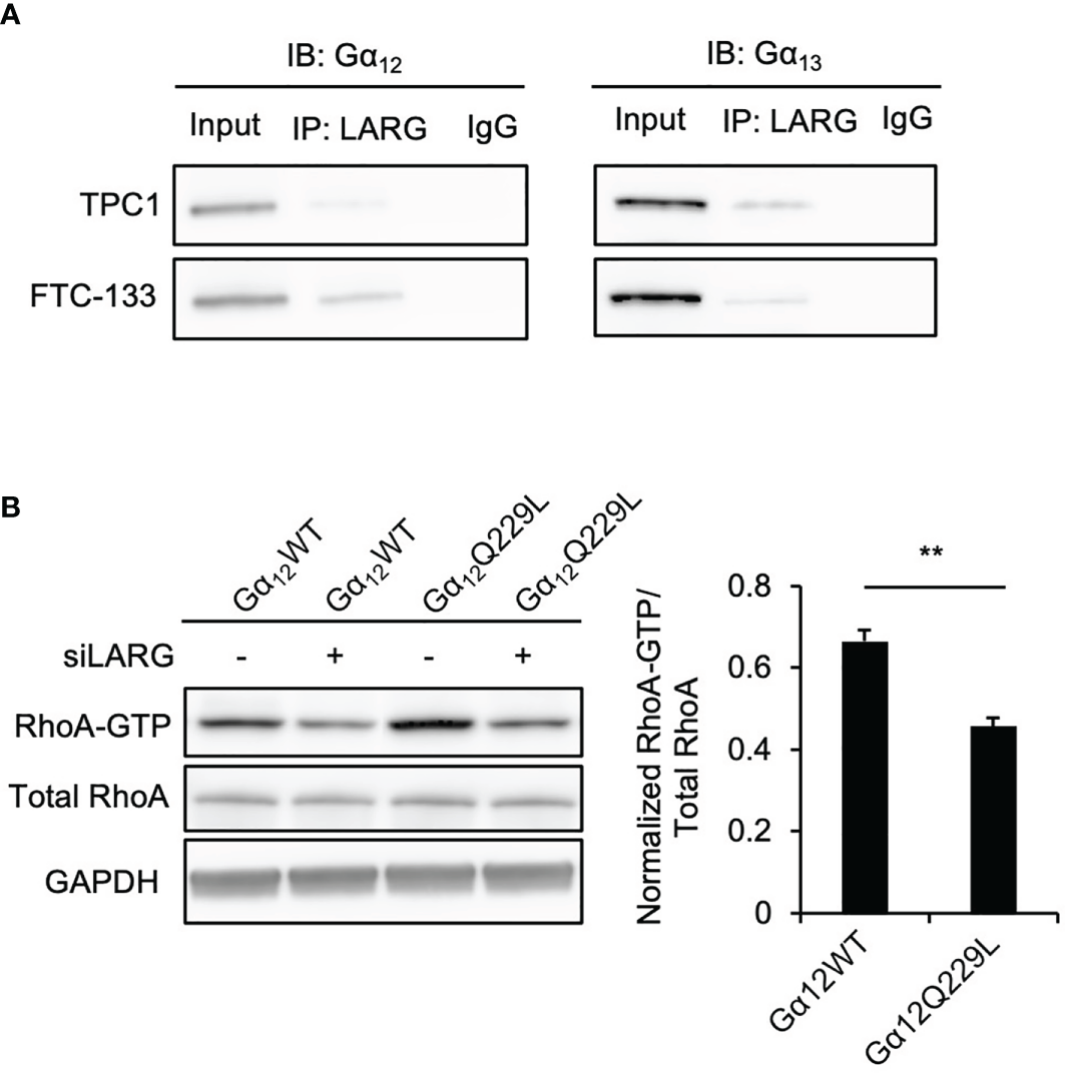
Gα_12/13_ interacts with LARG to activate RhoA in thyroid cancer cells. **(A)** Co-immunoprecipitation (IP) of Gα_12/13_ -LARG using lysates prepared from thyroid cancer cells. Input, 20 µg of total proteins from each cell line. The exposure time was 2 minutes. Left panel, Gα_12_ immunoblots from lysates immunoprecipitated with anti-LARG or control IgG in TPC1 and FTC-133 cells. Right panel, Gα_13_ immunoblots from lysates immunoprecipitated with anti-LARG or control IgG in TPC1 and FTC-133 cells. Data are representative of 3 sets of independent experiments. **(B)** Immunoblots of RhoA, and GAPDH from total cell lysates and RhoA after GST-Rhotekin pull-down (RhoA-GTP form) in TPC1 stably transfected with Gα_12_WT or Gα_12_Q229L then transiently transfected with siNT or siLARG. The bar graph represents relative active RhoA levels in TPC1 cells stably transfected with Gα_12_WT or Gα_12_Q229L and then transiently transfected with siLARG normalized to control. Data represent means ± SEM of 3 independent experiments. ^**^ *P* < 0.01.

RhoA activation is controlled by Rho guanine exchange factors (RhoGEFs) (23), and some GPCRs coupled to G proteins were found to interact with RhoGEFs, activating their downstream signaling. Our previous study revealed that the RhoGEF leukemia associated RhoA guanine exchange factor (LARG) is involved in thyroid tumorigenesis (20). To determine whether Gα_12/13_ can interact with LARG in thyroid cancer cells, we performed immunoprecipitation experiments. LARG was detected in Gα_12_ or Gα_13_ immunoprecipitates from whole-cell lysates of the thyroid cancer cell lines (**Figure 3A**). Then, to confirm that the Gα_12/13_-mediated increase in RhoA-GTP was dependent on LARG, we performed a RhoA activation assay in TPC1 cells stably transfected with Gα_12_Q229L or Gα_12_WT plasmid and then transiently transfected with siLARG or siNT. Again, Gα_12_Q229L-transfected cells showed a higher level of RhoA-GTP than control Gα_12_WT-transfected cells. After LARG knockdown, the RhoA-GTP level in all the cells decreased, and this decrease was more substantial in cells expressing Gα_12_Q229L. The RhoA-GTP level decreased by 0.67- and 0.46-fold in cells expressing Gα_12_WT and Gα_12_Q229L, respectively (*P*=0.004, **Figure 3B**). Taken together, our data indicate that TSH-TSHR couples with Gα_12/13_ and then interacts with LARG to activate RhoA, enhancing the migration of thyroid cancer cells.

### Gα_12/13_ Activation Is Associated With Thyroid-Specific Marker Loss and Decreased Iodide Uptake

**Figure 4:**
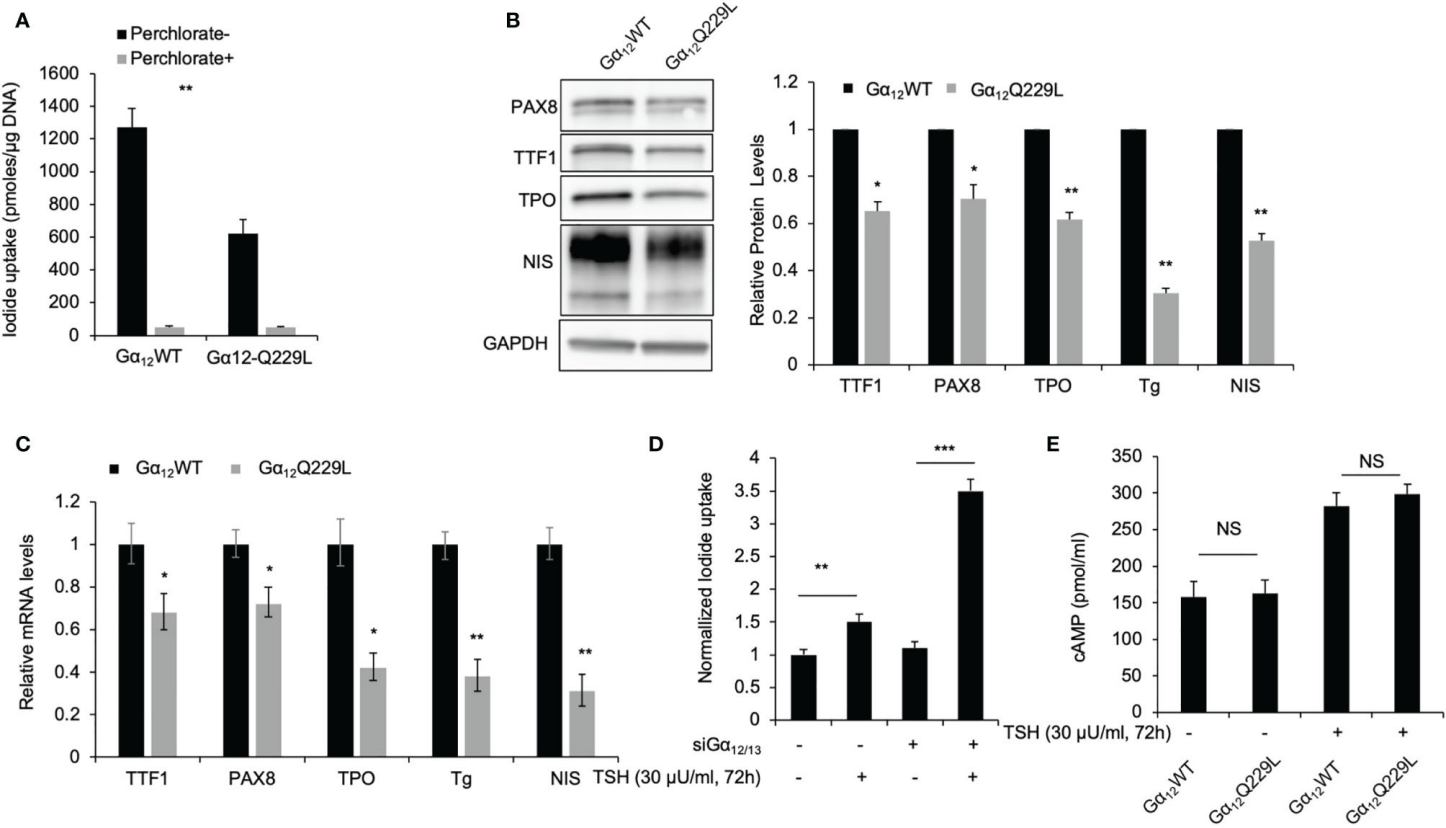
Constitutively active Gα_12/13_ is associated with thyroid specific maker loss and decreased iodide uptake. **(A)** Radioiodide uptake of PCCL3 cells stably transfected with Gα_12_Q229L or Gα_12_WT. Data represent means ± SEM of 3 independent experiments. **(B)** Immunoblots of TTF1, PAX8, TPO and NIS in PCCL3 cells stably transfected with Gα_12_Q229L or Gα_12_WT. GAPDH is used as a loading control for normalization. Bar charts, Relative TTF1, PAX8, TPO, Tg and NIS protein levels in PCCL3 cells stably transfected with Gα_12_Q229L or Gα_12_WT. Tg levels were detect by RIA. Data represent means ± SEM of 3 independent experiments. **(C)** Bar charts, Relative mRNA levels of TTF1, PAX8, TPO, Tg and NIS in PCCL3 cells stably transfected with Gα_12_Q229L or Gα_12_WT. Data represent means ± SEM of 3 independent experiments. **(D)** Normalized radioiodide uptake of PCCL3 cells transiently transfected with siGα_12/13_ or siNT then treated with 30μU/ml TSH or control. Data represent means ± SEM of 3 independent experiments. **(E)** Bar chart, cAMP levels in PCCL3 cells stably transfected with Gα_12_Q229L or Gα_12_WT incubated with 30 μU/ml TSH. Data are representative of 3 sets of independent experiments. ^*^ *P* < 0.05, ^**^ *P* < 0.01, ^***^ *P* < 0.001, NS, not significant.

TSH-TSHR signaling is known to increase iodide uptake in thyroid cells through the activation of Gα_s_-cAMP and downstream signaling; however, the role of Gα_12/13_ in iodide uptake remains elusive. Interestingly, we observed that transfection of the rat thyroid cell line PCCL3 with endogenous NIS expression with active Gα_12_Q229L significantly reduced iodide uptake (**Figure 4A**). Compared to control cells transfected with Gα_12_WT plasmid, cells transfected with Gα_12_Q229L plasmid showed decreased iodide uptake by approximately 49% (*P*=0.004). By qRT-PCR, Western blotting and Tg radioimmunoassay (RIA), we observed that both the mRNA and protein expression levels of the thyroid-specific genes TTF-1, PAX-8, Tg, TPO and NIS (**Figures 4B, C**) were significantly decreased in cells transfected with Gα_12_Q229L plasmid compared to cells transfected with Gα_12_WT plasmid. The protein levels were decreased 0.65-, 0.70-, 0.62-, 0.31- and 0.53-fold, respectively (all *P*<0.05). Similar results were observed in cells transfected with Gα_13_Q226L plasmid compared to control cells. Furthermore, TSH increased iodide uptake more significantly in cells in which Gα_12/13_ was knocked down compared to control cells; iodide uptake increased by 3.4- and 1.5-fold in cells transfected with siGα_12/13_ and siNT, respectively (*P*<0.001) (**Figure 4D**). These data indicate that the activation of Gα_12/13_ signaling might inhibit the expression of thyroid-specific molecules and reduce iodide uptake of thyroid cells. Then, we investigated cAMP levels in cells transfected with Gα_12_Q229L plasmid. Compared to control cells transfected with Gα_12_WT plasmid, cells transfected with Gα_12_Q229L plasmid did not show significantly changed cAMP levels, suggesting that Gα_12/13_ activation after thyroid-specific molecular expression and iodide uptake is independent of cAMP levels (**Figure 4E**).

### Gα_s_ Signaling Is Inhibited While Gα_12/13_ Activation in Thyroid Cancer Cells Undergoing Dedifferentiation

**Figure 5:**
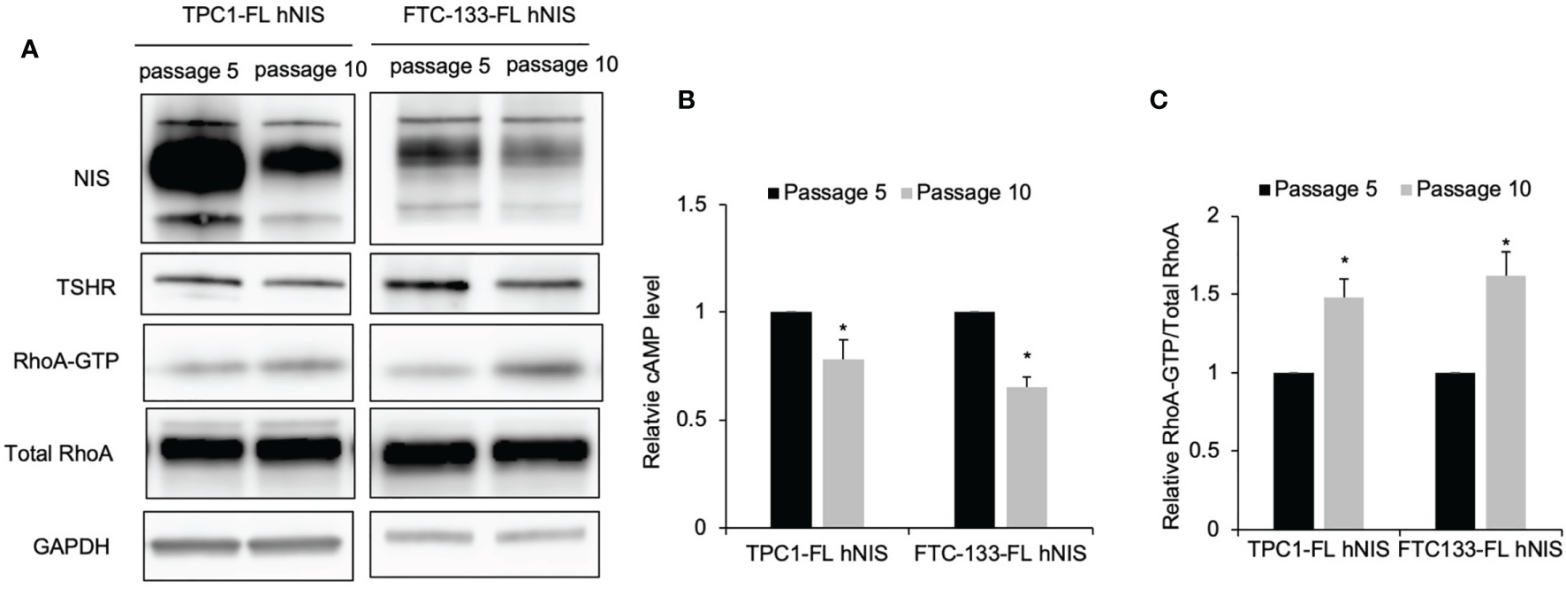
Gα_s_ signaling is inhibited while Gα_12/13_ activation in thyroid cancer cells undergoing dedifferentiation. **(A)** Immunoblot of NIS, TSHR, RhoA and GAPDH from total cell lysates and RhoA after GST-Rhotekin pull-down (RhoA-GTP form) in FTC-133-FL hNIS and TPC1-FL hNIS cells at passage 5 or passage 10. GAPDH is used as a loading control for normalization. Data are representative of 3 sets of independent experiments. **(B)** Bar chart, cAMP levels in FTC-133-FL hNIS and TPC1-FL hNIS cells at passage 5 or passage 10. Data are representative of 3 sets of independent experiments. **(C)** Bar graphs, normalized ratios of active RhoA relative to total RhoA levels. Data represent means ± SEM of 3 independent experiments. ^*^ *P* < 0.05.

Our previous studies revealed that thyroid cancer cells cultured *in vitro* underwent dedifferentiation exhibited decreased iodide uptake and expression of thyroid-specific molecules (11, 12). Since Gα_12/13_ and Gα_s_ signaling had the opposite effect in regulating iodide uptake, we then investigated the levels of RhoA activation associated with Gα_12/13_ and cAMP in our cell models of dedifferentiation compared to control cells. Again, our data confirmed that in TPC1 and FTC-133 cells with stable NIS expression, NIS and TSHR protein levels were lower in cells at passage 10 than in those at passage 5 (**Figure 5A**). Consistent with the change in TSHR level, in TPC1-FL hNIS and FTC-133-FL hNIS cells, the cAMP level was decreased by 0.78- and 0.65-fold, respectively (*P*=0.016 and 0.018, respectively; **Figure 5B**), in dedifferentiated cells compared to control cells. However, relative RhoA-GTP levels were higher in dedifferentiated cells than in control cells, with an increase of 1.48- and 1.62-fold, respectively (*P*=0.021 and 0.019, respectively; **Figure 5C**).

### PTEN/PI3K Facilitates Gα_12/13_ Signaling Activation Through Increased LARG Levels

**Figure 6:**
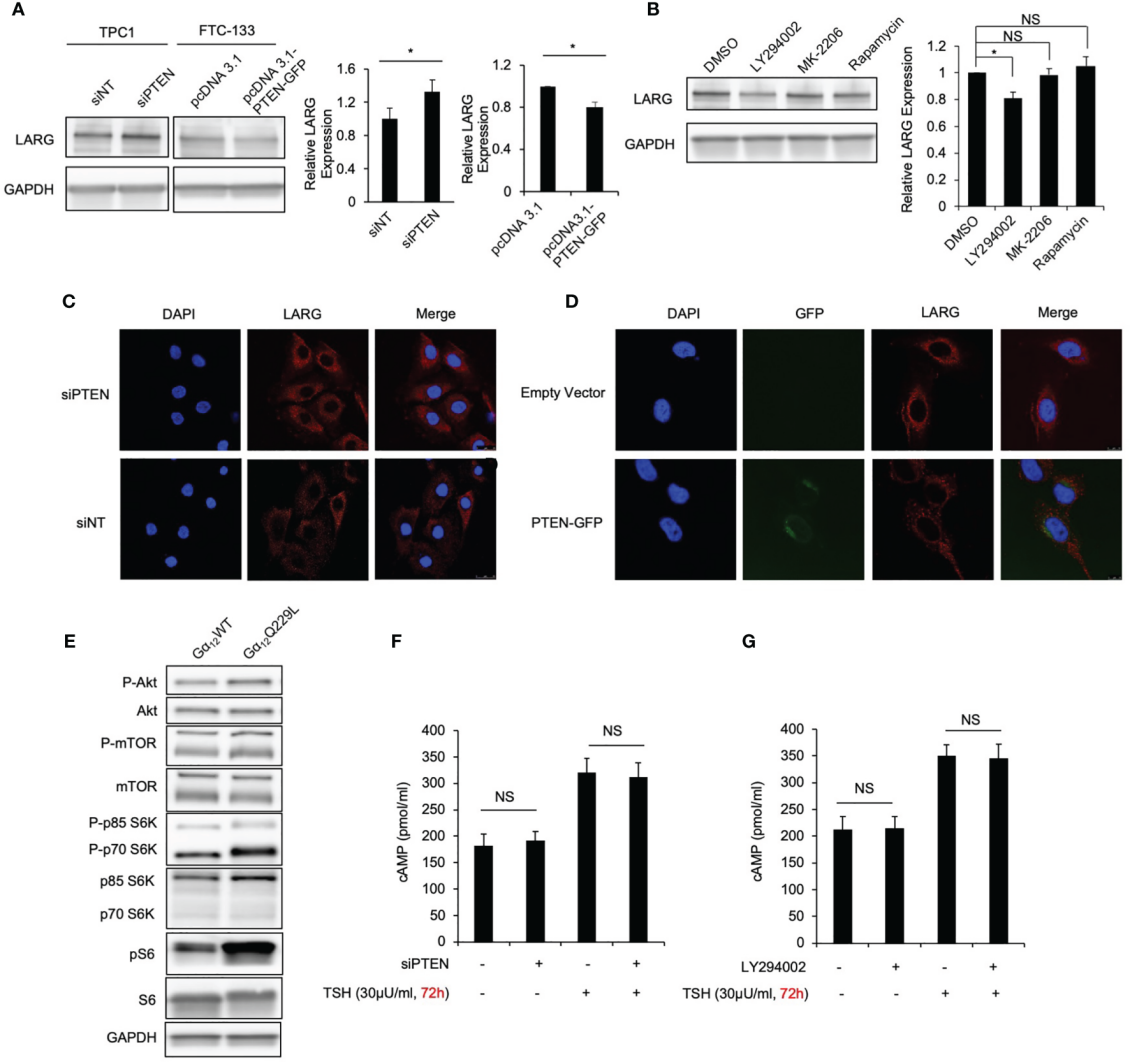
PTEN/PI3K is associated with LARG levels and submembrane localization in thyroid cancer cells. **(A)** Immunoblot of LARG using total lysate from TPC1 cells transiently transfected with siNT or siPTEN, and FTC-133 cells transiently transfected with empty vector or PTEN plasmid for 72 hours. GAPDH was used as a loading control. Bar chart shows the relative LARG protein levels in TPC1 after siPTEN knockdown compared to control, and in FTC-133 cells after PTEN transfection compared to control. Data represent means ± SEM of 3 independent experiments. **(B)** Immunoblot of LARG in TPC1 cells treated with LY294002, MK-2206, rapamycin or DMSO. Bar chart, LARG protein levels normalized to GAPDH. Data represent means ± SEM of 3 independent experiments. **(C)** Confocal microscopy of LARG immunofluorescence staining in TPC1 cells transiently transfected with siNT or siPTEN for 72 hours. Scale bar, 25 µm. Data are representative of 3 sets of independent experiments. **(D)** Confocal microscopy of LARG immunofluorescence staining in FTC-133 cells transiently transfected with PTEN-GFP or empty vector for 48 hours. Scale bar, 10 µm. Data are representative of 3 sets of independent experiments. **(E)** Immunoblot of phospho-Akt, Akt, phospho-mTOR, mTOR, phospho-p70 S6K, p70 S6K, phosphor-ribosomal S6, ribosomal S6 and GAPDH in TPC1 cells stably transfected with Gα_12_Q229L or Gα_12_WT. Data are representative of 3 sets of independent experiments. **(F)** Bar chart, cAMP levels in TPC1 cells transiently transfected with siNT or siPTEN, with or without TSH for 72 hours. Data represent means ± SEM of 3 independent experiments. **(G)** Bar chart, cAMP levels in TPC1 cells treated with PI3K inhibitor LY294002 or DMSO, with or without TSH for 72 hours. Data represent means ± SEM of 3 independent experiments. ^*^ *P* < 0.05, NS, not significant.

Our previous studies also revealed that PTEN/PI3K signaling affect the iodide uptake function of thyroid cancer cells. To test our hypothesis that PTEN/PI3K signaling is involved in crosstalk with the TSHR pathway in thyroid cancer cells, we first knocked down PTEN, the key regulator of PI3K signaling, by siRNA-mediated transfection in TPC1 and BCPAP cells. The loss of PTEN resulted in increased LARG levels in these thyroid cancer cell lines. The relative expression of LARG in TPC1 and BCPAP cells after PTEN knockdown was increased approximately 1.2- and 1.5-fold compared to that in control cells (*P*=0.015 and 0.009, respectively; **Figure 6A** and **Supplementary Figure S4**). In addition, the LARG level in FTC-133 cells (PTEN null) transiently transfected with PTEN plasmid was decreased 0.82-fold compared to that in cells transfected with the vector control (*P*=0.013; **Figure 6A**). Consistently, in TPC1 cells, treatment with the PI3K inhibitor LY294002 decreased LARG protein levels by 20% (*P*=0.025; **Figure 6B**). However, following treatment with the AKT inhibitor MK-2206 and the mTOR inhibitor rapamycin, LARG levels were not significantly difference compared to those in control cells, suggesting that LARG was regulated by the PI3K level. Moreover, by immunofluorescence, in TPC1 cells in which *PTEN* had been knocked down, LARG was predominately localized in the submembrane region (**Figure 6C**), where it can interact with Gα_12/13_. Consistently, in FTC-133 cells, *PTEN* transfection resulted in increased intracellular LARG localization (**Figure 6D**). Together, these data indicate that in thyroid cancer cells, PTEN/PI3K signaling activation facilitates Gα_12/13_-LARG-RhoA activation by increasing LARG levels and promoting the localization of LARG in the submembrane region.

Interestingly, in TPC1 cells, we observed that after active Gα_12_Q229L plasmid transfection, the expression levels of molecules downstream of PI3K (phospho-AKT, phospho-mTOR, phospho-P70 S6K and phosphor-ribosomal S6) were increased compared to those in control cells transfected with Gα_12_WT plasmid, suggesting that Gα_12/13_ activation is associated with the activation of molecules downstream of PI3K (**Figure 6E**).

### Activation of PI3K Signaling Decreases Iodide Uptake Independent of TSHR-Gα_s_-cAMP Signaling

Following the observation of crosstalk between PI3K and molecules downstream of TSHR-Gα_12/13_ signaling, we then investigated the effect of PI3K signaling on TSHR-Gα_s_-cAMP signaling. We did not observe a significant difference in measured intracellular cAMP levels between PTEN-knockdown TPC1 cells and control cells after siNT transfection treated with or without TSH (**Figure 6F**). Consistently, there was no significant difference in intracellular cAMP levels between cells treated with the PI3K inhibitor LY294002 and control cells (**Figure 6G**). Since our and others’ previous studies have shown that PI3K/AKT/mTOR signaling activation negatively regulates iodide uptake through regulating NIS expression and localization (20), we speculate that PI3K/AKT/mTOR signaling inhibits molecules downstream of Gα_s_ signaling.

## Discussion

Our observations reveal that TSH-TSHR increases thyroid cancer cell mobility through activating the Gα_12/13_ signaling pathway. The best known functions of TSH-TSHR signaling are its regulation of the proliferation and differentiation of thyroid follicular cells and iodide uptake and TH secretion by activation of the Gα_s_-cAMP-PKA pathway (24). In fact, as a GPCR, TSHR can couple to the members of all four G protein families (8). For example, TSHR couples to Gα_q_ and then activates the phospholipase C (PLC) pathway to stimulate iodination by stimulating H_2_O_2_ generation. However, the role of Gα_12/13_ in TSHR signaling has rarely been reported. Our data show that a high level of TSH increased the migration rate of thyroid cancer cells, which was abrogated after Gα_12/13_ knockdown. Furthermore, overexpression of the active mutant Gα_12_Q229L or Gα_13_Q226L also resulted in altered migration and dedifferentiation phenotypes. These data provide sufficient evidence that Gα_12/13_ is involved in the role of TSH-TSHR signaling in increasing the motility of thyroid cancer cells. Our data also suggest that TSHR signaling plays a protumorigenic role. TSHR expression has been reported to be associated with unfavorable prognosis in other cancers, such as liver cancer (25), but whether this is also the case in thyroid cancer remains unknown.

Our data demonstrated that TSH-TSHR-Gα_12/13_ signaling increases the migration of thyroid cancer cells through activating RhoA. RhoA activation, similar to the activation of other Rho family GTPases, plays an important role in enhancing cancer cell motility. Evidence supports the notion that in a number of cell lines, Gα_12/13_ can mediate signals through coupling GPCRs to Rho GTPase activation (26, 27). RhoA activation is controlled by RhoGEFs (23, 28). Our previous study revealed that a novel RhoGEF, LARG, is involved in tumorigenesis in thyroid cancer (24). Here, we observed that LARG interacts with Gα_12/13_ and activates RhoA in thyroid cancer cells and that this process is dependent on stimulation by high levels of TSH. This suggests that LARG mediates signal transduction from TSH-TSHR-Gα_12/13_ to RhoA activation, in turn increasing the migration of thyroid cancer cells.

Interestingly, while we observed that overexpression of the active mutant Gα_12_Q229L activated RhoA signaling to increase cell migration, it also resulted in decreased expression of the thyroid-specific molecules TTF1, PAX8, TPO, Tg and NIS and consistent iodide uptake in PCCL3 cells, independent of cAMP levels. Our data suggest that the activation of noncanonical TSHR-Gα_12/13_ signaling inhibits the expression of thyroid differentiation markers, thus promoting the dedifferentiation of thyroid cancer cells, although the mechanism warrants further study. One possible mechanism is the inhibited expression of thyroid-specific markers due to the downstream activation of RhoA. In fact, Medinaet et al. found that FRTL-5 cells expressing a constitutively active form of RhoA (RhoA QL) showed decreased levels of Tg and TTF-1 compared to those in cells expressing WT RhoA (29).

An investigation of how noncanonical Gα_12/13_ signaling is preferentially activated in thyroid cancer cells would be intriguing. Gain- or loss-of-function mutations in TSHR have been reported to be associated with the abnormal activation of downstream signaling (30, 31). For example, in transfected COS-7 cells, L653A TSHR expression was shown to reduce the downstream production of different G proteins to a different extent, as stimulated cAMP was decreased by 62%, while stimulated IP production was decreased 12% compared to that in cells expressing WT TSHR (32). This suggests that some mutations affect specific G protein signal transduction pathways. However, few TSHR mutations in thyroid cancer tissue have been reported. We speculate that crosstalk between pathways downstream of TSHR and other tumorigenesis signaling pathways plays an important role in aberrant TSHR signal transduction.

PI3K/AKT/mTOR signaling activation, which is negatively regulated by the tumor suppressor gene PTEN, enhances cell proliferation and migration in multiple cancer cells, including thyroid cancer cells (16). Importantly, PI3K/AKT/mTOR signaling is also associated with decreased iodide uptake in thyroid cancer cells (17). We investigated the relationship between PI3K/AKT/mTOR signaling and TSHR signaling and found that PI3K/AKT/mTOR signaling activation does not affect pathways upstream of Gα_s_ and Gα_12/13_. However, PI3K/AKT/mTOR signaling exhibited crosstalk with pathways downstream of Gα_12/13_ by increasing LARG levels. Interestingly, the loss of PTEN was also found to promote LARG localization in the submembrane region, particularly under the migration leading edge, where it can interact with and activate RhoA to mediate cell migration. We also observed that active mutant Gα_12_Q229 overexpression resulted in the activation of molecules downstream of PI3K signaling, further proving that these two signaling pathways form a network to regulate the migration and differentiation of thyroid cancer cells.

We also carried out experiments to explore the mechanism by which Gα_12/13_ activation collaborates with PI3K signaling to decrease thyroid-specific gene expression and iodide uptake. We observed that PI3K signaling inhibition by the pharmacologic drug LY294002, PTEN knockdown by siRNA and constitutively active Gα_12/13_ overexpression did not change cAMP levels, which normally accumulate due to Gα_s_ and activate PKA to regulate thyroid-specific gene transcription. However, some groups have reported that treatment with PI3K inhibitors, as well as inhibitors of molecules downstream of PI3K, such AKT and mTOR, increases thyroid-specific gene expression and iodide uptake in thyroid cells (19, 33). Based on our observation that constitutively active Gα_12/13_ decreased thyroid-specific protein expression and activated PI3K signaling, we speculate that Gα_12/13_ collaborates with PI3K/AKT/mTOR signaling to regulate pathways downstream of the cAMP pathway. However, the exact target of this regulation warrants further study.

It has been reported that BCPAP cells harbor *BRAF^V600E^* mutation (34). However, the effect of TSH on migration phenotype is similar as TPC1 cells which have wild type *BRAF*. Interestingly, we observed the effect of TSH is more significant in FTC-133 cells which PTEN is null. Thus, whether BRAF-MAPK signaling is associated with Gα_12/13_ signaling warrant further study. Some research has reported *BRAF* mutation associated with MAPK activation affect Gα_S_ signaling to inhibit iodine uptake of thyroid cancer cells (35). We speculate that PI3K signaling play more import roles in the transform from well differentiated in to poor- or un-differentiated thyroid cancer especially the migration phenotype.

**Figure 7:**
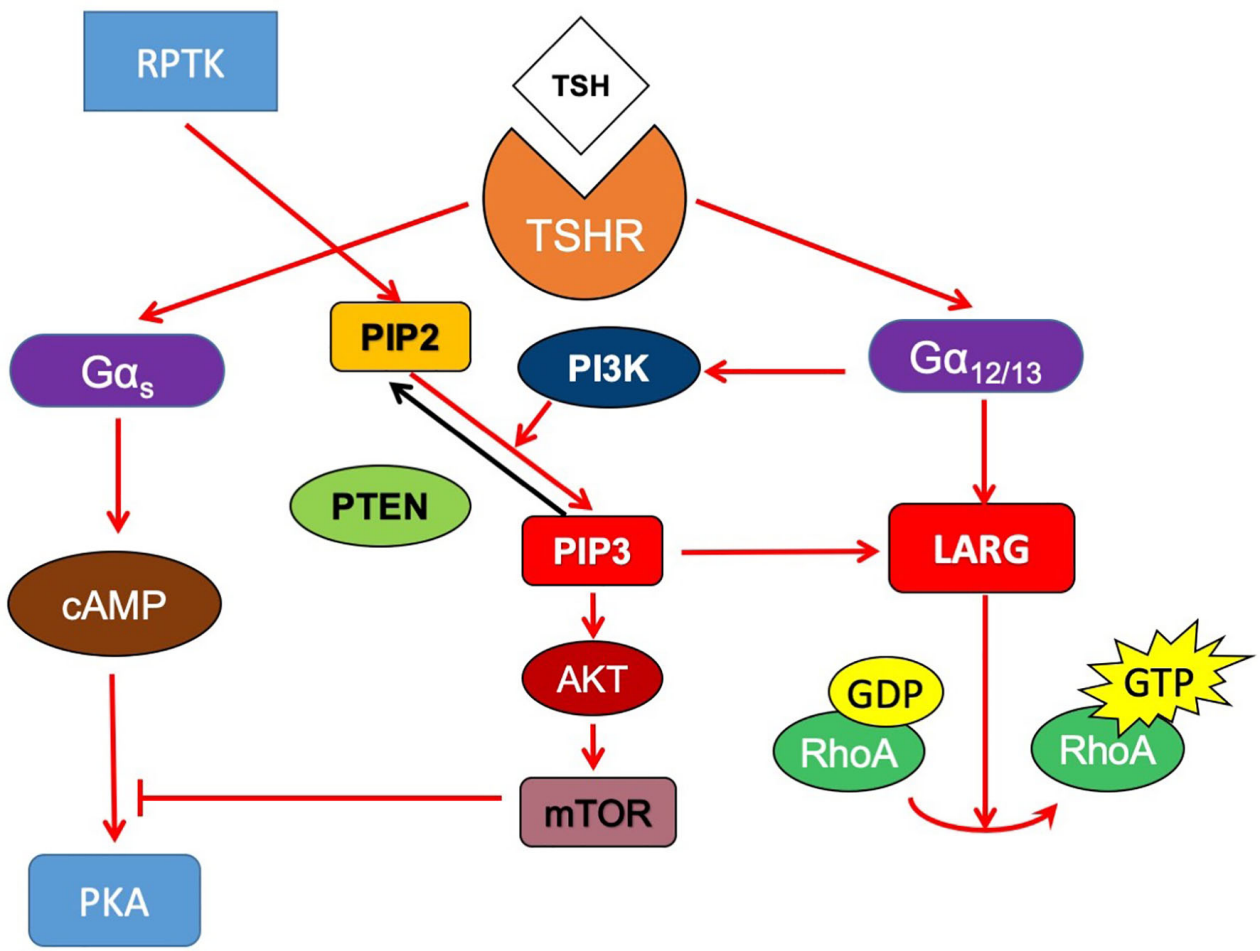
Proposed signaling model of crosstalk between TSHR and PI3K signaling pathway. In thyroid cancer cells, TSHR couples to Gα_12/13_, interacts with LARG and then activates RhoA to increase migration. PI3K signaling activation increased LARG levels in cancer cells and altered its localization to facilitate the interaction of LARG with Gα_12/13_ and enhance TSHR-Gα_12/13_ signaling. Gα_12/13_ signaling also might participate with PI3K to inhibit the pathways downstream Gα_s_ signaling.

In summary, we found that in thyroid cancer cells, TSHR couples to Gα_12/13_, interacts with LARG and then activates RhoA to increase migration. In addition, constitutively active Gα_12/13_ expression decreased thyroid-specific gene expression and iodide uptake in thyroid cells. PI3K signaling activation increased LARG levels in cancer cells and altered its localization to facilitate the interaction of LARG with Gα_12/13_ and enhance TSHR-Gα_12/13_ signaling. Gα_12/13_ signaling participates with PI3K to inhibit the pathways downstream Gα_s_ signaling (**Figure 7**). Together, our data suggest that in thyroid cancer, noncanonical activation of TSH-TSHR signaling through Gα_12/13_ plays an important role in cell motility and the dedifferentiation of thyroid cancer. These findings have broader implications as they are relevant to the function of TSHR in thyroid cancer and understanding of the dedifferentiation of thyroid cancer in parallel with invasion.

## Data Availability Statement

The original contributions presented in the study are included in the article/**Supplementary Material**. Further inquiries can be directed to the corresponding author.

## Author Contributions

Conception and design: FF. Development of methodology: HH and FF. Acquisition of data: HH. Analysis and interpretation of data: HH and SW. Writing, review and/or revision of the manuscript: FF, HH, SW, and HW. Administrative, technical, or material support: FF and HW. Study supervision: FF and HW. All authors contributed to the article and approved the submitted version.

## Funding

This study was funded by the National Natural Science Found of China (grant 81974269) and Biomedical-engineering Cross Fund of Shanghai Jiao Tong University (grant YG2019QNA39).

## Conflict of Interest

The authors declare that the research was conducted in the absence of any commercial or financial relationships that could be construed as a potential conflict of interest.

## Publisher’s Note

All claims expressed in this article are solely those of the authors and do not necessarily represent those of their affiliated organizations, or those of the publisher, the editors and the reviewers. Any product that may be evaluated in this article, or claim that may be made by its manufacturer, is not guaranteed or endorsed by the publisher.
