# Correction: Crosstalk between abnormal TSHR signaling activation and PTEN/PI3K in the dedifferentiation of thyroid cancer cells

**DOI:** 10.3389/fonc.2026.1897172

**Published:** 2026-07-17

**Authors:** Fang Feng, Huiqin Han, Shuqi Wu, Hui Wang

**Affiliations:** 1 Department of Nuclear Medicine, Xinhua Hospital, Shanghai Jiao Tong University, School of Medicine, Shanghai, China; 2 Shanghai Mental Health Center, Shanghai Jiao Tong University, School of Medicine, Shanghai, China

**Keywords:** differentiated thyroid carcinoma, thyrotropin receptor, radioiodine, dedifferentiation, migration

There was a mistake in **Figure 1** as published. Due to an oversight, one image of TPC-1 in **Figure 1B** was inadvertently not updated. The corrected **Figure 1** appears below.

**Figure 1:**
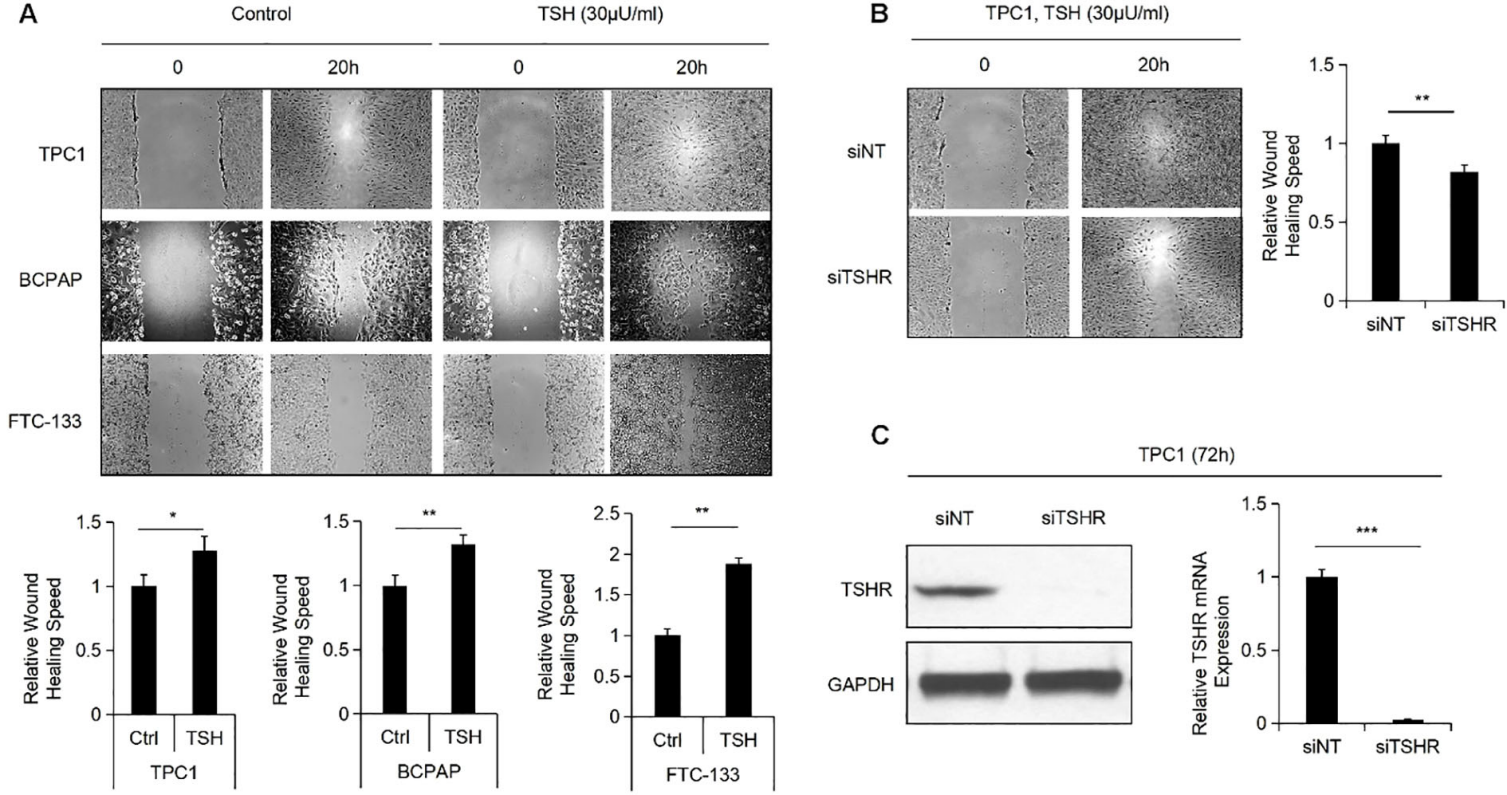
TSH increasing RhoA activation and cell migration is Gα12/13 dependent. **(A)** Immunoblots of RhoA and GAPDH from total cell lysates and RhoA after GST-Rhotekin pull-down (RhoA-GTP form) in TPC1, BCPAP and FTC-133 cells. Bar graphs, normalized ratios of active RhoA relative to total RhoA levels. Data represent means ± SEM of 3 independent experiments. **(B)** Wound-healing assay for TPC1 cells with treatment of 30 μU/mL TSH and 30μM Y27632 or control. Bar graph Data represents relative wound healing rate of TPC1 cells with treatment of 30 μU/mL TSH and 30μM Y27632 normalized with control. Immunoblots of phosphor-coffin and GAPDH in TPC1 cells incubated with 30 μU/mL TSH and 30μM Y27632 for 72 hours. and Data represent means ± SEM of 3 independent experiments. **(C)** Immunoblots of RhoA, Gα12 and GAPDH from total cell lysates and RhoA after GST-Rhotekin pull-down (RhoA-GTP form) in TPC1, BCPAP and FTC-133 transiently transfected with non-targeting siRNA (siNT) or Gα12 and Gα13 siRNA (si Gα12/13) with treatment of 30μU/mL TSH. GAPDH is used as a loading control for normalization. The bar graph represents normalized ratios of active RhoA relative to total RhoA levels. **(D)** Immunoblots of RhoA, Gα12 and GAPDH from total cell lysates and RhoA after GST-Rhotekin pull-down in TPC1 stably transfected with Gα12WT or Gα12Q229L. Bar graph, normalized ratios of active RhoA relative to total RhoA levels. *P 0.05, **P 0.01, ***P 0.001.

The original version of this article has been updated.

