# Supplementary material for: Crosstalk Between Abnormal TSHR Signaling Activation and PTEN/PI3K in the Dedifferentiation of Thyroid Cancer Cells
Source: Front Oncol. 2021 Sep 28;11:718578. doi: 10.3389/fonc.2021.718578 (PMC8506026; doi:10.3389/fonc.2021.718578)
Supplement: Supplementary file 1 [file DataSheet1.pdf]

Supplementary Material

Supplementary Figures

Fig. S1

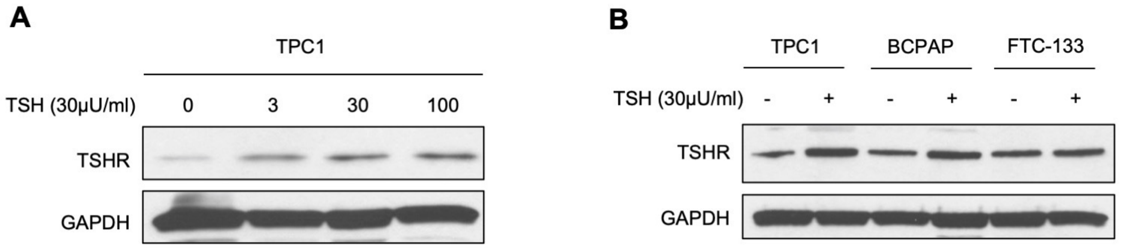

Supplementary Figure 1. TSH induced TSHR expression in thyroid cancer cell: A. Immunoblots of TSHR in TPC1 cells treated with 0, 3, 30 or 100 μU/ml TSH for 48 hours. Control wells was added with HBSS of the same volume. GAPDH was set as the loading control. B. Immunoblots of TSHR in TPC1, BCPAP and FTC-133 cells treated with 30 μU/ml TSH for 48 hours. Control wells was added with HBSS of the same volume. GAPDH was set as the loading control.

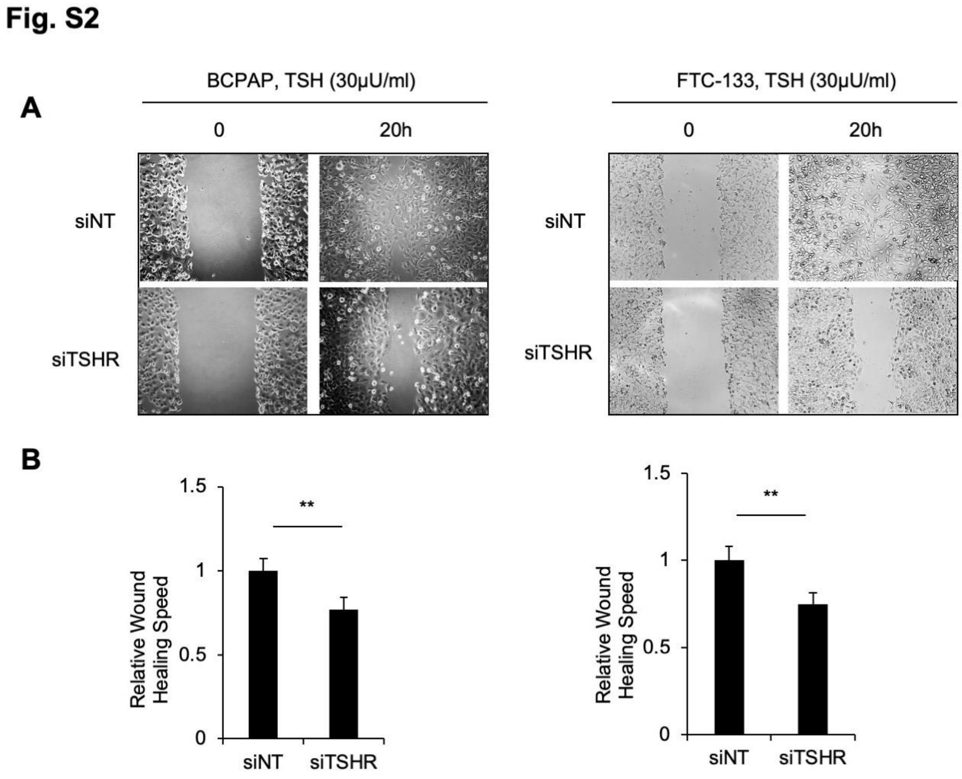

Supplementary Figure S2. TSH-TSHR increase cell migration in thyroid cancer cells: **A**. Wound-healing assay for BCPAP and FTC-133 cells incubated with 30 μU/mL TSH transiently transfected with non-targeting siRNA control (siNT) or TSHR siRNA (siTSHR) for 72 hours. **B**. Bar graph represents relative wound healing rate of BCPAP and FTC-133 cells after siTSHR knockdown normalized with control. Data represent means ± SEM of 3 independent experiments. $^{**}P$<0.01.

Fig. S3

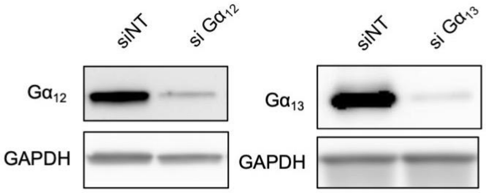

Supplementary Figure S3. $G\alpha_{12}$ and $G\alpha_{13}$ was confirmed to be knocked down by Western blot: Left panel, immunoblot of $G\alpha_{12}$ and GAPDH in TPC1 cells transiently transfected with siNT or si$G\alpha_{12}$ for 72 hours. Right panel, immunoblot of $G\alpha_{13}$ and GAPDH in TPC1 cells transiently transfected with siNT or si$G\alpha_{13}$ for 72 hours.

Fig. S4

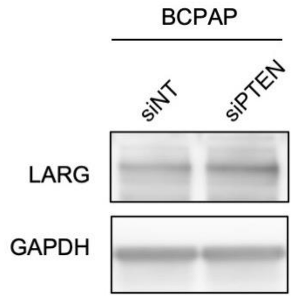

Supplementary Figure S4. LARG expression level increased in BCPAP cells after siPTEN knockdown: Immunoblot of LARG and GAPDH in BCPAP cells transiently transfected with siNT or siPTEN for 72 hours.
